# Asthma–Chronic Obstructive Pulmonary Diseases Overlap Syndrome Increases the Risk of Incident Tuberculosis: A National Cohort Study

**DOI:** 10.1371/journal.pone.0159012

**Published:** 2016-07-22

**Authors:** Jun-Jun Yeh, Yu-Chiao Wang, Chia-Hung Kao

**Affiliations:** 1 Ditmanson Medical Foundation Chia-Yi Christian Hospital, Chiayi, Taiwan; 2 Chia Nan University of Pharmacy and Science, Tainan, Taiwan; 3 Meiho University, Pingtung, Taiwan; 4 Management Office for Health Data, China Medical University Hospital, Taichung, Taiwan; 5 College of Medicine, China Medical University, Taichung, Taiwan; 6 Graduate Institute of Clinical Medical Science, College of Medicine, China Medical University, Taichung, Taiwan; 7 Department of Nuclear Medicine and PET Center, China Medical University Hospital, Taichung, Taiwan; University of Manitoba, CANADA

## Abstract

**Purpose:**

The association between asthma–chronic obstructive pulmonary diseases (COPD) overlap syndrome (ACOS) and tuberculosis (TB) has yet to be studied.

**Methods:**

The newly diagnosed TB patients (age > 20 y) treated from January 2000 to December 2008 were included (ACOS cohort, n = 10 751; non-ACOS cohort, n = 42 966). The non-ACOS cohort involved patients with confirmed absence of ACOS. We calculated incidence rate ratios (IRRs) for TB in the ACOS and non-ACOS cohorts by using poisson regression analysis. Cox proportional hazards regression models were used to determine the adjusted HR (aHR) for TB in the ACOS cohort compared with the non-ACOS cohort.

**Results:**

The aHR for TB was 2.41 (95% confidence interval [CI], 2.19–2.66) in the ACOS cohort. The TB risk was significantly higher in the ACOS cohort than in the non-ACOS cohort when stratified by age, sex, comorbidities, and atopy. Within the ACOS cohort, the aHR was higher among patients receiving SABAs+SAMAs, LABAs+LAMAs, and ICSs (aHR [95% CI]: 3.06 [2.75–3.41], 3.68 [2.93–4.61], and 2.79 [1.25–6.22], respectively; all *P* < .05). Furthermore, patients with more than 15 outpatient visits and hospitalizations per year demonstrated the highest aHR (8.09; 95% CI, 6.85–9.56).

**Conclusions:**

ACOS cohort potentially develop incident TB, regardless of the age,sex, comorbidities and atopy; even without receiving the inhalers.This risk is higher, especially in the ACOS cohort have a high frequency of medical services or receiving the inhalers such as SABAs+SAMAs, LABAs+LAMAs and ICSs.

## Introduction

Asthma–chronic obstructive pulmonary diseases (COPD) overlap syndrome (ACOS) is clinically defined as representing either a hybrid of eosinophilic bronchiolitis [1] (asthma, typically childhood-onset, Th2-mediated inflammation, and induced sputum eosinophilia ≥ 3%) [2] and neutrophilic bronchiolitis (COPD or adult-onset asthma and Th1-mediated inflammation) [3,4] or independent clinical entities [3,4]. A previous study demonstrated that the association of the Th2 signature with increased severity and asthma-like features (eg, a favorable corticosteroid response) in ACOS suggests that Th2 inflammation is crucial for disease identification in COPD subsets with an unclear clinical history of asthma [5]. Tai et al revealed that children with severe asthma are at an increased COPD risk [6]. According to these findings, ACOS can be identified by its features shared between asthma and COPD. ACOS is being increasingly recognized [7], and its prevalence reportedly increases with age. In a 5-year follow-up study, the incidence of acute respiratory events was higher in the ACOS cohort than in the COPD cohort [8]; therefore, ACOS is a burden on hospital staff globally [9],[10].

The pharmacotherapy of ACOS [8] is similar to that of COPD and asthma [11]. The medications normally prescribed for COPD can potentially be useful in patients with critical asthma syndrome, particularly those with ACOS [12]. In the phenotype-based pharmacotherapeutic approach, bronchodilators alone are considered in patients with the nonfrequent exacerbator phenotype of COPD [13], whereas a combination of bronchodilators and inhaled corticosteroids (ICSs) [14] is considered in patients with ACOS[15] or with the moderate-to-severe exacerbator phenotype of COPD [16].

Patients with COPD may have a high frequency of longer hospital stays, thus increasing their susceptibility to nosocomial tuberculosis (TB) [17]. Anemia [18], pneumonia[18] and hypoalbuminemia [19] are predisposing factors of readmission for COPD. Patients with COPD are at a high risk of nutritional deficiency, which is associated with declines in respiratory function, lean body mass, strength, and immune function [20]. These elements [21] are also critical risk factors for TB [22,23]. Meanwhile, the smoking–related diseases (e.g. hypertension, hyperlipidemia, diabetes, pneumonia, alcohol-related illnesses, stroke, ischemic heart disease) [24], cancer [25], postinflammatory fibrosis (PPF) [26] and human immunodeficiency virus (HIV) infection [22] were potential risk factors of the TB also [25]

The ACOS may consider as a different entity [27] in the chronic airway limitations diaeases [28] and the relationship of this disorder with the TB has not been reported in the English literature. Therefore, in this study, we hypothesized that ACOS may play a role in the development of incident TB, and we tested this hypothesis by conducting a cohort study involving the general population of Taiwan.

## Methods

### Data Source

The National Health Insurance (NHI) program of Taiwan was established in March 1995. It consolidates 13 insurance programs by the Taiwan Department of Health, with a coverage rate of approximately 99% of the population of Taiwan since 2000. All claims data from the NHI program, including beneficiary registry, disease records, and other medical services, are collected in the National Health Insurance Research Database (NHIRD). We used the Longitudinal Health Insurance Database 2000 (LHID2000) for establishing our study cohort. LHID2000 comprises claims data collected from one million people randomly selected from the total insurant population during 1996–2011. Taiwan’s NHI constructed this disease record system on the basis of the International Classification of Diseases, Ninth Revision, Clinical Modification (ICD-9-CM). To ensure the privacy of the insurants, the Taiwan National Health Research Institutes uses only scrambled and anonymous numbers to indicate the files of the insurants.

### Ethics Statement

The NHIRD encrypts patient personal information to protect privacy and provides researchers with anonymous identification numbers associated with relevant claims information, including sex, date of birth, medical services received, and prescriptions. Therefore, patient consent is not required to access the NHIRD. This study was approved to fulfill the condition for exemption by the Institutional Review Board (IRB) of China Medical University (CMUH104-REC2-115). The IRB also specifically waived the consent requirement.

### Study Population

**Fig 1:**
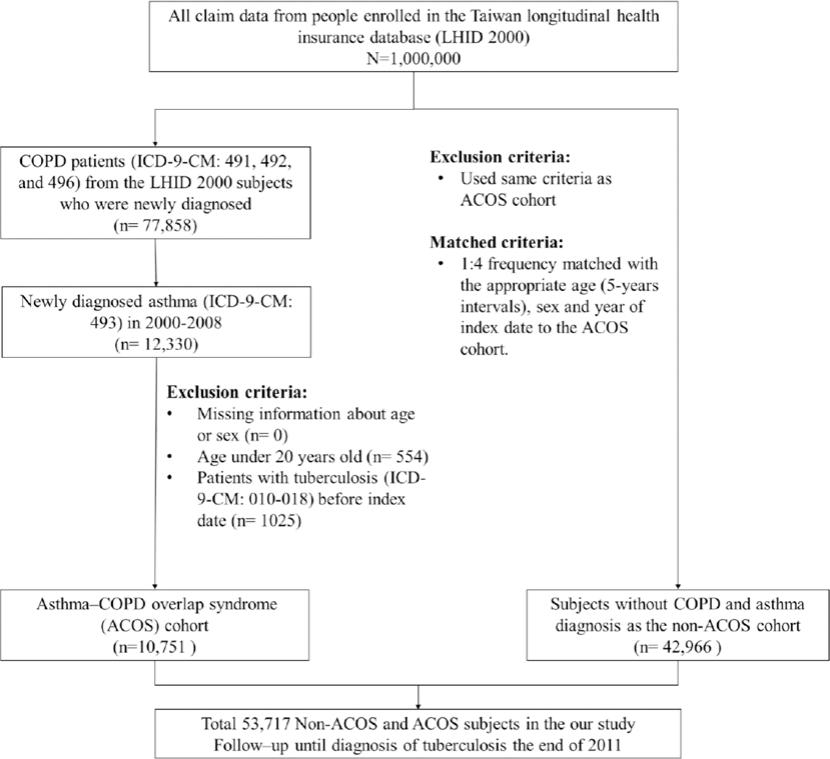
Process of selecting subjects for study cohorts.

We used a retrospective population-based cohort study design. Fig 1 shows the flow chart for selecting the study population. ACOS patients were aged >20 y and treated from January 2000 to December 2008. The ACOS cohort comprised patients with COPD (ICD-9-CM codes: 491, 492, and 496) and concurrent physician-diagnosed asthma patients (ICD-9-CM code: 493) [8]. The date of asthma diagnosis was defined as the index date. In this study, the pure COPD cohort exclude asthma component and the pure asthma cohort exclude COPD component, respectively[29]. Only, a physician diagnosis of asthma and COPD [27] in the same patient [29] enrolled in the ACOS cohort.[28] Therefore, the ACOS cohort have the nearly same number of the syndrome [29,30] with asthma and COPD (persistent airflow limitation with several features usually associated with asthma and several features usually associated with COPD), this definition in line with the syndromic approach the chronic airway limitations in Global Initiative for Asthma (GINA) report. ACOS cohort is therefore identified by the features it shares with both asthma and COPD) [11].The non-ACOS cohort contained patients with confirmed absence of ACOS. For each patient in the ACOS cohort, 4 controls were selected using the frequency-matching method on the basis of age (at 5-y intervals), sex, and the year of the index date. We excluded patients diagnosed with TB (ICD-9-CM codes: 010–018) before the index date in both cohorts. The follow-up period was from the index date until withdrawal from the NHI, development of TB, or December 31, 2011.

The confounding factors, including age, sex, and TB-associated comorbidities, were controlled in the analysis model for adjustment. Patients with comorbidities were defined as those with a disease history before the baseline. The TB-associated comorbidities included allergic rhinitis (AR; ICD-9-CM code: 477), atopic dermatitis (AD; ICD-9-CM code: 691), allergic conjunctivitis (AC; ICD-9-CM codes: 372.05, 372.10, and 37214), hypertension (ICD-9-CM codes: 401–405), hyperlipidemia (ICD-9-CM code: 272), diabetes (ICD-9-CM code: 250), alcohol-related illnesses (ICD-9-CM codes: 291, 303, 305, 571.0, 571.1, 571.2, 571.3, and 790.3), pneumonia (ICD-9-CM codes: 480–487), ischemic heart disease (IHD; ICD-9-CM codes: 410–414), stroke (ICD-9-CM codes: 430–438)[24], postinflammatory pulmonary fibrosis (PPF; ICD-9-CM codes: 515), cancer (ICD-9-CM codes: 140–208), and HIV infection (ICD-9-CM codes: 795.71, V08, 042, 079.53).

We also considered the medicine treatment effect of the following drugs and their combinations on the association between ACOS and TB: inhaled short-acting β-agonists (SABAs) alone, inhaled short-acting muscarinic antagonists (SAMAs) alone, a combination of inhaled SABAs and SAMAs, inhaled long-acting β-agonists (LABAs) alone, inhaled long-acting muscarinic antagonists (LAMAs) alone, and ICSs.

### Statistical Analyses

The chi-square test was used to analyze the differences in categorical variables (sex and comorbidities) between the ACOS and non-ACOS cohorts, with the results presented as the numbers and percentages for these variables. The continuous variable (age) was analyzed using the Student *t* test, with the results presented as mean and standard deviation (SD). The incidence density of TB was calculated as the number of TB events divided by the sum of observation time (per 1000 person-y). In both cohorts, the incidence rate ratios (IRRs) for TB and their 95% confidence intervals (95% CIs) were measured for these variables by using Poisson regression analysis. The cumulative incidence curves for the 2 cohorts were plotted using the Kaplan–Meier method, and the differences between the curves were analyzed using the log-rank test. To elucidate the TB risk, crude hazard ratios (HRs) and adjusted HRs (aHRs) and their 95% CIs were estimated using Cox proportional hazards regression models. Furthermore, the multivariable Cox proportional hazards regression model was used to estimate the TB risk on the basis of the drugs used for treatment; the model was also used to elucidate the association between frequency of outpatient visits and hospitalizations (per year) and TB risk among patients with ACOS. A 2-sided *P* value of < .05 was considered significant. SAS 9.4 software (SAS Institute, Cary, NC, USA) was used for data management and statistical analyses, whereas the incidence curves were plotted using R software (R Foundation for Statistical computing, Vienna, Austria).

## Results

**Fig 2:**
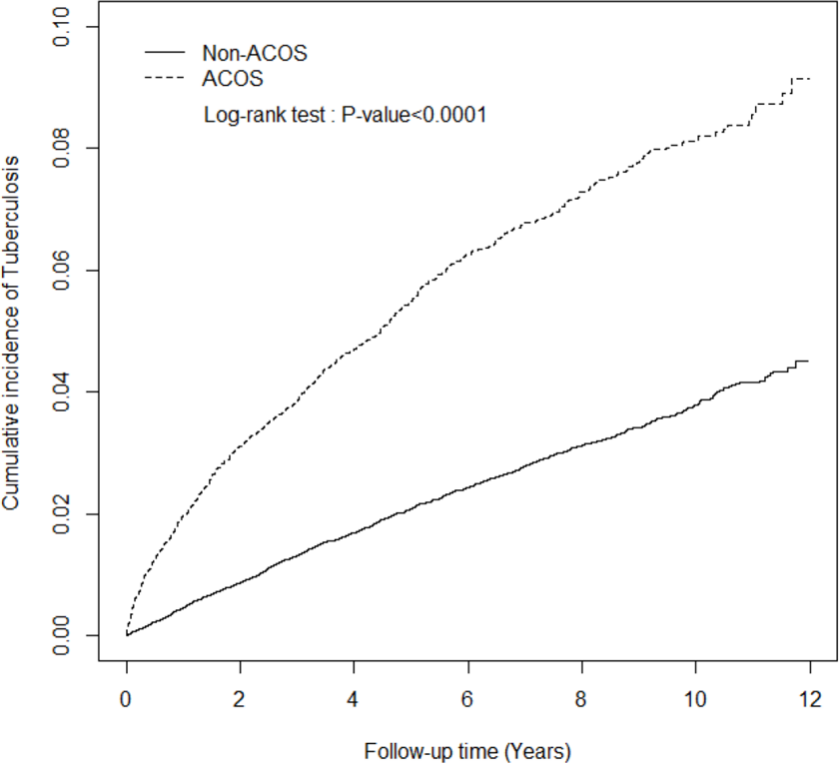
The cumulative incidence of tuberculosis in asthma–COPD overlap syndrome (ACOS) (dashed line) and non-ACOS cohorts (solid line).

This study included a total of 10 751 patients with ACOS and 42 966 patients without ACOS (Table 1). After frequency matching for age and sex, we determined no significant differences between the ACOS and non-ACOS cohorts. The mean age of the patients in the ACOS cohort was 64 years (SD, 14.4; *P* = .26), with most of them aged >65 years (55.1%). Nearly 55% of the patients with ACOS were men. Moreover, patients with ACOS were more likely to have TB-associated comorbidities than were those without ACOS (all *P* < .0001, chi-square test) except for cancer and HIV infection. The cumulative incidence of TB in the ACOS cohort was significantly higher than that in the non-ACOS cohort (*P* < .0001, log-rank test; Fig 2).

**Table 1:** Comparison of demographics and comorbidity between ACOS and non-ACOS cohorts.

	ACOS				
	No (N = 42966)		Yes (N = 10751)		
	n	%	n	%	*p*-value
**Sex**					0.97
Women	19161	44.6	4792	44.6	
Men	23805	55.4	5959	55.4	
**Age, year**					0.99
20–35	1560	3.63	390	3.63	
35–65	17740	41.3	4435	41.3	
≥65	23666	55.1	5926	55.1	
Mean (SD) ^#^	64.2 (14.4)		64.6 (14.4)		0.26
**Comorbidity**					
AR	4852	11.3	3244	30.2	< .0001
AD	1079	2.51	441	4.10	< .0001
AC	9399	21.9	3080	28.6	< .0001
Alcohol-related illness	592	1.38	329	3.06	< .0001
Diabetes	9568	22.3	2982	27.7	< .0001
Hypertension	21166	49.3	6836	63.6	< .0001
Hyperlipidemia	11603	27.0	3698	34.4	< .0001
Pneumonia	8397	19.5	4161	38.7	< .0001
IHD	12042	28.0	4742	44.1	< .0001
Stroke	8724	20.3	3072	28.6	< .0001
PPF	80	0.19	57	0.53	< .0001
Cancer	1476	3.44	380	3.53	0.61
HIV infection	8	0.02	2	0.02	0.99

Chi-square test

^#^ Student’s t-test.

ACOS, asthma–COPD overlap syndrome; AR, allergic rhinitis; AD, atopic dermatitis; AC, allergic conjunctivitis; IHD, ischemic heart disease; PPF, postinflammatory pulmonary fibrosis.

During follow-up, 688 TB events (incidence rate: 9.64 per 1000 person-y) occurred in the ACOS cohort, whereas 1202 (incidence rate: 3.99 per 1000 person-y) occurred in the non-ACOS cohort. The IRR of TB in the ACOS cohort was 2.42 compared with the non-ACOS cohort (95% CI, 2.29–2.55). After controlling for age, sex and history of each comorbidity, we determined that the TB risk in the ACOS cohort was 2.41-fold higher than that in the non-ACOS cohort (aHR [95% CI], 2.41 [2.19–2.66]). Compared with those without ACOS, both men and women patients with ACOS showed increased TB risks (aHR [95% CI]: 2.26 [2.02–2.54] and 2.90 [2.38–3.53], respectively). The age-stratified analysis also revealed similar results: the TB risk was higher among patient with ACOS than among those without ACOS, regardless of the age: 20–65-year group (aHR [95% CI], 2.90 [2.35–3.60]) and ≥65-year group (aHR [95% CI], 2.21 [1.97–2.47]). Regarding patients without any comorbidity, the ACOS cohort demonstrated a 3.49-fold higher TB risk than did the non-ACOS cohort (95% CI, 2.61–4.66). For patients with at least one comorbidity, the aHR of TB in the ACOS cohort was 2.36 greater than that in the non-ACOS cohort (95% CI, 2.13–2.61). Compared with atopy patients without ACOS, the TB risk was nearly 2-fold higher in atopy patients with ACOS (95% CI, 1.61–2.25) (Table 2).

**Table 2:** Incidence and adjusted hazard ratio of tuberculosis stratified by sex, age, and comorbidity between ACOS and non-ACOS cohorts.

	ACOS							
		No			Yes		Compared to non-ACOS cohort	
Variables	Event	PY	Rate	Event	PY	Rate	IRR* (95% CI)	Adjusted HR^†^ (95% CI)
**Overall**	1202	301225	3.99	688	71365	9.64	2.42(2.29–2.55)***	2.41(2.19–2.66)***
**Sex**								
Women	276	139241	1.98	189	33389	5.66	2.86(2.63–3.10)***	2.90(2.38–3.53)***
Men	926	161984	5.72	499	37975	13.1	2.30(2.15–2.46)***	2.26(2.02–2.54)***
**Age, year**								
20–65	227	149780	1.52	177	36817	4.81	3.17(2.92–3.44)***	2.90(2.35–3.60)***
≥65	975	151445	6.44	511	34547	14.8	2.30(2.15–2.46)***	2.21(1.97–2.47)***
**Comorbidity**								
No	238	89635	2.66	57	6816	8.36	3.15(2.74–3.62)***	3.49(2.61–4.66)***
Yes	964	211590	4.56	631	64549	9.78	2.15(2.02–2.27)***	2.36(2.13–2.61)***
**Atopic syndrome**								
No	851	218178	3.90	433	36738	11.8	3.02(2.83–3.23)***	2.73(2.42–3.08)***
Yes	351	83047	4.23	255	34626	7.36	1.74(1.60–1.90)***	1.91(1.61–2.25)***

ACOS, asthma–COPD overlap syndrome; Atopic syndromes including allergic rhinitis, atopic dermatitis, and allergic conjunctivitis; PY, person-year; Rate, incidence rate (per 1,000 person-years); IRR*, incidence rate ratio; Adjusted HR^†^: multiple analysis including sex, age, comorbidity of allergic rhinitis, atopic dermatitis, allergic conjunctivitis, alcohol-related illness, diabetes, hypertension, hyperlipidemia, pneumonia, ischemic heart disease, stroke, postinflammatory pulmonary fibrosis, cancer, and HIV infection.

*p<0.05

***p<0.001.

Table 3 presents data for various risk factors for TB analyzed in this study. Men patients were at a higher risk of developing TB than were women patients (aHR [95% CI], 2.28 [2.05–2.53]). The TB risk increased with age (aHR [95% CI], 3.11 [2.77–3.51] for those aged ≥65 y). The TB risk was higher in patients who had comorbidity of diabetes (aHR [95% CI], 1.14 [1.02–1.27]), pneumonia (aHR [95% CI], 1.45 [1.31–1.60]), stroke (aHR [95% CI], 1.19 [1.07–1.33]), and PPF (aHR [95% CI], 2.54 [1.53–4.23]), respectively. The low risk of TB was presented in AR patients (aHR [95% CI], 079 [0.69–0.90]), AC patients (aHR [95% CI], 0.85 [0.76–0.96]) and hyperlipidemia patients (aHR [95% CI], 0.74 [0.66–0.83]). There was no statistically significant to increase TB risk in patient with AD (aHR [95% CI], 1.24 [0.98–1.59]), alcohol-related illness (aHR [95% CI], 1.09 [0.78–1.53]), hypertension (aHR [95% CI], 1.05 [0.94–1.17]), IHD (aHR [95% CI], 1.02 [0.92–1.13]), cancer (aHR [95% CI], 0.99 [0.77–1.28]) and HIV infection (aHR [95% CI], 3.16 [0.45–2.25]).

**Table 3:** The adjusted hazard ratio of tuberculosis in different risk factors.

Variables	N	Event	Crude HR (95% CI)	Adjusted HR (95% CI)
**ACOS**				
No	42966	1202	1.00	1.00
Yes	10751	688	2.40(2.19–2.64)***	2.36(2.14–2.60)***
**Sex**				
Women	23953	465	1.00	1.00
Men	29764	1425	2.62(2.36–2.91)***	2.28(2.05–2.53)***
**Age groups, years**				
20–65	24125	404	1.00	1.00
≥65	29592	1486	3.61(3.23–4.03)	3.11(2.77–3.51)***
**Comorbidity**				
**AR**				
No	45621	1621	1.00	1.00
Yes	8096	269	0.99(0.87–1.12)	0.79(0.69–0.90)***
**AD**				
No	52197	1822	1.00	1.00
Yes	1520	68	1.50(1.18–1.91)**	1.24(0.98–1.59)
**AC**				
No	41238	1483	1.00	1.00
Yes	12479	407	0.99(0.89–1.11)	0.85(0.76–0.96)**
**Alcohol-related illness**				
No	52796	1855	1.00	1.00
Yes	921	35	1.32(0.94–1.84)	1.09(0.78–1.53)
**Diabetes**				
No	41167	1396	1.00	1.00
Yes	12550	494	1.29(1.16–1.42)***	1.14(1.02–1.27)*
**Hypertension**				
No	25715	724	1.00	1.00
Yes	28002	1166	1.67(1.52–1.83)	1.05(0.94–1.17)
**Hyperlipidemia**				
No	38416	1412	1.00	1.00
Yes	15301	478	0.88(0.80–0.98)*	0.74(0.66–0.83)***
**Pneumonia**				
No	41159	1272	1.00	1.00
Yes	12558	618	1.85(1.68–2.04)***	1.45(1.31–1.60)***
**IHD**				
No	36933	1158	1.00	1.00
Yes	16784	732	1.56(1.42–1.71)***	1.02(0.92–1.13)
**Stroke**				
No	41921	1336	1.00	1.00
Yes	11796	554	1.77(1.60–1.95)***	1.19(1.07–1.33)***
**PPF**				
No	53580	1875	1.00	1.00
Yes	137	15	4.37(2.63–7.26)***	2.54(1.53–4.23)***
**Cancer**				
No	51861	1827	1.00	1.00
Yes	1856	63	1.24(0.97–1.60)	0.99(0.77–1.28)
**HIV infection**				
No	53707	1889	1.00	1.00
Yes	10	1	3.59(0.51–25.3)	3.16(0.45–22.5)

ACOS, asthma–COPD overlap syndrome; AR, allergic rhinitis; AD, atopic dermatitis; AC, allergic conjunctivitis; Adjusted HR: multiple analysis including sex, age groups, comorbidity of AR, AD, AC, alcohol-related illness, diabetes, hypertension, hyperlipidemia, pneumonia, ischemic heart disease, stroke, postinflammatory pulmonary fibrosis, cancer, and HIV infection.

*p<0.05

**p<0.01

***p<0.001.

Table 4 presents the comparison of the TB risk among patients with and without ACOS with regard to the treatment. Patients with ACOS not receiving SABAs+SAMAs, LABAs+LAMAs, or ICSs showed a higher TB risk than did those without ACOS (aHR [95% CI]: 1.29 [1.05–1.60], 2.14 [1.91–2.40], and 2.15 [1.90–2.43], respectively). ACOS patients receiving SAMAs alone had a 1.95-fold higher TB risk than did those without ACOS (95% CI, 1.08–3.54). ACOS patients receiving SABAs+SAMAs presented a 3.06-fold higher TB risk than did those without ACOS (95% CI, 2.75–3.41). Compared with those without ACOS, the aHRs (95% CIs) for TB were 2.89 (2.37–3.35), 2.66 (1.84–3.85), and 3.68 (2.93–4.61) in ACOS patients receiving LAMAs alone, LABAs alone, and LAMAs+LABAs, respectively. ACOS patients receiving ICSs had a 2.79-fold higher TB risk than did those without ACOS (95% CI, 1.25–6.22).

**Table 4:** Adjusted hazard ratio of tuberculosis found in the follow-up period associated with ACOS and prescriptions of SABAs, SAMAs, LAMAs, LABAs, or ICSs.

**Variables**	**N**	**Event**	**Rate**	**Adjusted HR (95% CI)**
**Non-ACOS cohort**	42966	1202	3.99	1.00
**ACOS cohort**				
Without SABAs and SAMAs	3501	94	3.68	1.29(1.05–1.60)*
Only used SABAs	1568	54	4.74	1.56(1.18–2.05)***
Only used SAMAs	207	11	7.71	1.95(1.08–3.54)*
Used SABAs and SAMAs	5475	529	16.03	3.06(2.75–3.41)***
Without LAMAs and LABAs	8021	428	8.19	2.14(1.91–2.40)***
Only used LAMAs	287	29	14.79	2.89(2.37–3.35)***
Only used LABAs	1888	150	11.33	2.66(1.84–3.85)***
Used LAMAs and LABAs	555	81	20.66	3.68(2.93–4.61)***
Without ICSs	6697	376	8.79	2.15(1.90–2.43)***
Used ICSs	4054	312	10.92	2.79(1.25–6.22)*

ACOS, asthma–COPD overlap syndrome; LABA, long-acting b-agonist; LAMA, long-acting muscarinic antagonist; SABA, short-acting b-agonist; SAMA, short-acting muscarinic antagonist; ICS, inhaled corticosteroid; Adjusted HR: multiple analysis including sex, age, comorbidity of allergic rhinitis, atopic dermatitis, allergic conjunctivitis, alcohol-related illness, diabetes, hypertension, hyperlipidemia, pneumonia, ischemic heart disease, stroke, postinflammatory pulmonary fibrosis, cancer, and HIV infection.

*p<0.05

***p<0.001.

The association of TB risk with the frequency of outpatient visits and hospitalizations per year because of COPD or asthma exacerbation is presented in Table 5. When this frequency increased, the aHR for TB among the patients with ACOS also increased from 1.24 (95% CI, 1.05–1.46) for those with 3 or more visits per year to 8.09 (95% CI, 6.85–9.56) for those with more than 15 visits (*P* < .0001), compared with the non-ACOS cohort.

**Table 5:** The adjusted hazard ratio of tuberculosis associated with number of outpatient visits and hospitalizations per year due to COPD or asthma exacerbation.

**Variables**	**N**	**Event**	**Rate**	**Adjusted HR (95% CI)**
**Non-ACOS cohort**	42966	1202	3.99	1.00
**Number of outpatient visits and hospitalizations per year in ACOS cohort (n = 10751)**				
≤ 3	5709	173	4.00	1.24(1.05–1.46)**
3–7	2299	166	11.1	2.46(2.09–2.91)***
7–15	1778	178	17.7	3.17(2.70–3.73)***
>15	965	171	56.4	8.09(6.85–9.56)***
*p-value for trend*				< .0001

ACOS, asthma–COPD overlap syndrome; Adjusted HR: multivariable analysis including sex, age, comorbidity of allergic rhinitis, atopic dermatitis, allergic conjunctivitis, alcohol-related illness, diabetes, hypertension, hyperlipidemia, pneumonia, ischemic heart disease, stroke, postinflammatory pulmonary fibrosis, cancer, and HIV infection.

**p<0.01

***p<0.001.

## Discussion

A crucial finding of this study is that patients with ACOS cohort have a higher TB risk than do those without ACOS, regardless of age, sex, or comorbidities. The clinical manifestations of ACOS include in the elderly population [31] and young adult [32], early-onset asthma following steroid treatment [33], and immunocompromised condition because of corticosteroid usage at a relatively higher-than-prescribed dose [34]. These manifestations are potential risk factors for TB. Frequent intensive care unit hospitalization [8] because of recurrent and severe exacerbation of ACOS also increases the nosocomial TB risk. Therefore, even in the absence of comorbidities, ACOS is primarily a critical risk factor for TB.

The AD increased risk of the TB in the previous studies [35], probably because of immunodeficiency [35]. The current study determined that the TB risk did’nt increase in the presence of the AD. By contrast, other atopies such as AR and AC reduced the TB risk. Moreover, the relationships between allergic diseases and TB reported by von Mutius et al [36] and Flohr et al [37] are controversial. The relationship of atopic disease with the TB warrant further investation.

We also observed that the smoking-related diseases such as diabetes were associated with the TB risk. This finding is consistent with that of Lin et al. [38] Meanwhile, pneumonia [39] and PPF [26] increased risk of the TB in line with the previous study [26,39].Cardiovascular diseases (e.g.stroke) [8,24] are critical risk factors leading to hospitalization, thus increasing the susceptibility of patients with ACOS to nosocomial TB. By contrast, hyperlipidemia reduces the TB risk; in hyperlipidemia, the cholesterol levels are high [40], and this may prevent malnutrition, reducing the TB risk. The other smoking related-diseases (e.g.alcohol-relaed illness, hypertension,IHD) and malnutrition-related diseases (e.g. cancer, HIV) didn’t increase the TB risk.The relationship of the smoking-related diseases and the malnutrition-related diseases with the TB risk in the ACOS cohort warrant further investigation.

In the current study, patients with ACOS receiving SABAs+SAMAs or LABAs+LAMAs demonstrated higher TB risk than did those receiving SABAs, SAMAs, LABAs, or LAMAs alone. During acute exacerbation of ACOS, patients receive combined therapy without ICSs [41],—either SABAs+SAMAs or LABAs+LAMAs [42]—but it does not reduce the criticality of such acute respiratory events [8], thus leading to frequent hospitalizations and increasing the patients’ susceptibility to nosocomial TB infection.

We observed that patients receiving ICSs for the severe phenotype of ACOS had a higher TB risk [43]. A high dosage or long-term use [44] of ICSs for the severe phenotype of ACOS leads to a high frequency of hospitalizations, contributing to a higher nosocomial TB risk. Patients with ACOS receiving a combined therapy containing ICSs have the highest TB risk [25]. This is corroborated by our result that higher the frequency of outpatient visits and hospitalizations, higher the TB risk.

In the Lee et al study[45], compared the 23 594 COPD cases with the 47 188 non-COPD control subjects based on the NHIRD, they found that ICSs were not independent risk factors for TB. In versa, a recent Chung et.al study based on the NHIRD also, [25] compared the 8091 TB patients (including the 1017 asthma patients and 2342 COPD patients) with the 32 364 non-TB patients indicated that long-term use of ICSs is associated with a 2.04-fold increased risk of developing TB. Our study agree this conclusion. This imply that the patients in the ACOS cohort [31] sharing both the asthma component and COPD component[3] in this disorder, and this cohort may be a different entity[46] in the chronic airway limitation[28].Therefore, the ICSs paly a different role on the risk of the TB in the different cohort.

Finally, even ACOS patients not receiving any inhaled bronchodilator or ICSs demonstrated high TB risk. ACOS is a combination of eosinophilic and neutrophilic bronchiolitis [47], and its asthma component is associated with mycoplasma pneumonia[48]. Therefore, airway remodeling [49] associated with epithelial desquamation as well as submucosal lymphocyte infiltration [5] may result in TB infection. In addition, high intake of oral bronchodilators [45] and high frequency of the admissions[8,34] may be factors causing the immunocompromised condition among patients with ACOS. This supports our finding that these factors in combination also contribute to the TB risk,even without receiving the inhalers.

A retrospective cohort study revealed that patients with ACOS have a significantly lower health-related quality of life, with more frequent and severe acute exacerbations, despite a relatively younger age and lower burden of cigarette smoking [32]. Similarly, our patients with ACOS appeared more likely to have a considerably lower quality of life and more frequent acute exacerbation than did patients with COPD alone. This assumption is supported by the lung tissue destruction, emphysema [50], and air trapping observed on the chest CT of patients with ACOS. Our study results may alert clinical physicians regarding the early detection of ACOS among patients with an obstructive airway disease such as COPD [8,31,51]. In addition, our patients with ACOS receiving monotherapy (SABAs, SAMAs, LABAs, LAMAs, or ICSs) or combined therapy (SABAs+SAMAs or LABAs+ LAMAs) showed higher frequency of acute respiratory distress events than did those without ACOS [8]. In this study, the TB risk and frequency of outpatient visits and hospitalizations were higher in the ACOS cohort receiving the inhaled bronchodilators (SABAs+SAMAs and LABAs+LAMAs) or ICSs than in the non-ACOS cohort.Therefore, the clinical physician should be aware of the incident TB among the ACOS cohort in either the receiving the inhalers or not receiving the inhalers at the same time, regardless of the age, sex and comorbidities.

### Limitations

The definition and treatment of ACOS varies in the literature. In addition, we did not include systemic steroids, antibiotics, or oral bronchodilators, such as leukotrine modifiers, phosphodiesterase-4 inhibitors, and theophylline in the analysis. Meanwhile, smoking habits, malnutrition status and environment factors were not analyzed. We only analyze the smoking–related diseases (e.g.hypertension, hyperlipidemia, diabetes, alcohol-related illnesses, pneumonia, stroke, IHD) and malnutrition-related diseases (e.g.cancer, HIV) for avoiding the bias. These confounding factors are essential and warrant investigation.

### Strengths

This study included a cohort representing the general population. In Taiwan, before prescribing an inhaler, patients must be screened through the pulmonary function test (PFT), and a public nurse respiratory educates them regarding its use and avoiding the environmental factor, assess the nutrition status and evaluates the immune status.For diagnosing asthma [52] and COPD[53], the patient history, clinical manifestations, pulmonary function [53], and thoracic imaging indicate the diseases; in addition, consensus of the chest physician, rheumatologist, and immunologist[54,55] is required. After diagnosis, the inhaler [56] is prescribed under the strict policies of the NHI member. Meanwhile, under the policy of establishing a physician centered model for releasing long-term prescription and escalating public medical knowledge to prevent over-use of National Insurance Resource, we need follow up the PFT [57]. In Cheng et al study, the COPD patients received the chest-X-ray (84.7%), PFT (58.4%), and a computed tomography scan (39.4%) in accordance with this finding [58]. Hence, even the PFT were unaviable in the NHIRD. Based on these strategies, the nearly over half of all patients in the ACOS cohort receiving the PFT (e.g. coexistence of increased variability of airflow in a patient with incompletely reversible airway obstruction) [51] at least 2 times under the services of multidisciplinary team. The chest physician [54,55] must be trained by the Taiwan Society of Pulmonary and Critical Care Medicine, Taiwan Association of Asthmatics, Taiwan Association of Chronic Obstructive Pulmonary Disease, or the Chinese of Society of Immunology. Similarly, the coding of TB requires a consensus of well-trained chest specialists and infection specialists as well as review against the Centers for Disease Control and Prevention criteria. In this study, we addressed the majority of drugs used for treating ACOS. We also analyzed atopies, which play a crucial role in ACOS development. Therefore, we exclude the TB in the the patients have pure the asthmatic components or pure the COPD components. These main strengths of our study aid in discriminating patients with COPD or asthma from ACOS patients for estimating susceptibility to TB and clarifying the association of ACOS with incident TB.

## Conclusions

ACOS cohort potentially develop incident TB, regardless of the age,sex, comorbidities, even without receiving the inhalers.This risk is higher, especially in the ACOS cohort have a high frequency of medical services or receiving the inhalers such as SABAs+SAMAs, LABAs+LAMAs and ICSs.

## Abbreviations

ACOS: Asthma–chronic obstructive pulmonary disease overlap syndrome
aHR: adjusted hazard ratio
CI: confidence interval
COPD: chronic obstructive pulmonary diseases
ICSs: inhaled corticosteroids
NHIRD: National Health Insurance Research Database
NHI: National Health Insurance
NHRI: National Health Research Institutes
LHID 2000: Longitudinal Health Insurance Database 2000
TB: tuberculosis
